# Supplementary material for: Effect of the COVID-19 Pandemic on Physical Activity and Sedentary Behavior in Older Adults
Source: J Clin Med. 2022 Mar 12;11(6):1568. doi: 10.3390/jcm11061568 (PMC8952771; doi:10.3390/jcm11061568)
Supplement: Supplementary file 1 [file jcm-11-01568-s001.zip › jcm-1623206-supplementary.pdf]

Impact of COVID-19 on Changes to Physical Activity and Sedentary Behaviors

Please think about all physical activity you did over the ***3 months before the COVID-19 pandemic*** (from December 2019 to February 2020), the ***first 3 months of the COVID-19 pandemic*** (from March 2020 to May 2020), and ***the past 3 months*** (from April 2021 to June 2021), including activities at work (occupational), as part of your house and yard work (household), to get from place to place (transportation), and in your spare time (leisure-time). People tend to over-report their activity, so please answer ***as accurately as possible***. There is no right or wrong answer.

	**3 months before the COVID-19 pandemic** (Dec 2019 – Feb 2020)	**First 3 months of the COVID-19 pandemic** (March 2020 – May 2020)	**Past 3 months** (April 2021 – June 2021)
Think about the time you spent **sitting** at work, at home, in a car, or during leisure time. (*Example: eating meals, sitting at a desk, driving a car, reading, watching TV or videos, using computer or smartphone, or playing computer games).*			
Total sitting time **on a weekday** (Monday-Friday)?	________ hours/day	________ hours/day	________ hours/day
Total sitting time **on a weekend day** (Saturday & Sunday)?	________ hours/day	________ hours/day	________ hours/day
Think about all the time you spent **walking**, including at work and home, walking to travel from place to place, or any other walking that you might do solely for recreation, sport, exercise, or leisure.			
Total walking time **on a weekday** (Monday – Friday)?	___ hour(s) ___ min /day	___ hour(s) ___ min /day	___ hour(s) ___ min /day
Total walking time **on a weekend day** (Saturday & Sunday)?	___ hour(s) ___ min /day	___ hour(s) ___ min /day	___ hour(s) ___ min /day
Think about all the **moderate physical activity** you did that caused only light sweating or a slight/moderate increase in breathing or heart rate. Do not include walking. (*Examples: slow bicycling, doubles tennis, golf, house cleaning such as vacuuming or sweeping, washing car, general gardening, or lawn mowing).*			
How many total times per week did you do these activities?	________ times/week	________ times/week	________ times/week
Average duration in each time?	________ minutes	________ minutes	________ minutes
Think about all the **vigorous physical activity** you did that caused heavy sweating or large increases in breathing or heart rate. Do not include walking or moderate physical activity. (*Examples: running, fast bicycling, singles tennis, basketball, football, hiking uphill, or heavy gardening like digging).*			
How many total times per week did you do these activities?	________ times/week	________ times/week	________ times/week
Average duration in each time?	________ minutes	________ minutes	________ minutes
Think about ***any*** physical activity specifically designed to **strengthen your muscles**. *(Examples: weight training, push-ups/sit-ups, or carrying heavy loads.)*			
How many total times per week did you do these activities?	________ times/week	________ times/week	________ times/week
Average duration in each time?	________ minutes	________ minutes	________ minutes
